# Central carbon metabolism remodeling as a mechanism to develop drug tolerance and drug resistance in *Mycobacterium tuberculosis*

**DOI:** 10.3389/fcimb.2022.958240

**Published:** 2022-08-22

**Authors:** Hyungjin Eoh, Rachel Liu, Juhyeon Lim, Jae Jin Lee, Philip Sell

**Affiliations:** Department of Molecular Microbiology and Immunology, Keck School of Medicine, University of Southern California, Los Angeles, CA, United States

**Keywords:** drug tolerance, drug resistance, tuberculosis, metabolomics, catalytic shift

## Abstract

Suboptimal efficacy of the current antibiotic regimens and frequent emergence of antibiotic-resistant *Mycobacterium tuberculosis* (Mtb), an etiological agent of tuberculosis (TB), render TB the world’s deadliest infectious disease before the COVID-19 outbreak. Our outdated TB treatment method is designed to eradicate actively replicating populations of Mtb. Unfortunately, accumulating evidence suggests that a small population of Mtb can survive antimycobacterial pressure of antibiotics by entering a “persister” state (slowly replicating or non-replicating and lacking a stably heritable antibiotic resistance, termed drug tolerance). The formation of drug-tolerant Mtb persisters is associated with TB treatment failure and is thought to be an adaptive strategy for eventual development of permanent genetic mutation-mediated drug resistance. Thus, the molecular mechanisms behind persister formation and drug tolerance acquisition are a source of new antibiotic targets to eradicate both Mtb persisters and drug-resistant Mtb. As Mtb persisters are genetically identical to antibiotic susceptible populations, metabolomics has emerged as a vital biochemical tool to differentiate these populations by determining phenotypic shifts and metabolic reprogramming. Metabolomics, which provides detailed insights into the molecular basis of drug tolerance and resistance in Mtb, has unique advantages over other techniques by its ability to identify specific metabolic differences between the two genetically identical populations. This review summarizes the recent advances in our understanding of the metabolic adaptations used by Mtb persisters to achieve intrinsic drug tolerance and facilitate the emergence of drug resistance. These findings present metabolomics as a powerful tool to identify previously unexplored antibiotic targets and improved combinations of drug regimens against drug-resistant TB infection.

## Highlights

- Unlike other omics techniques, metabolomics reveals the biochemical state of Mtb as metabolites are the final products of enzymatic, posttranslational, allosteric, and environmental integration.
- High-persistence forms of Mtb have complicated chemotherapeutic options because they are likely to evolve drug-resistant Mtb strains.
- Mtb in a persistent state shifts its metabolic networks to mitigate the production of antibiotic-induced reactive oxygen species and accompanied cellular damage, whereby Mtb persisters evade mycobactericidal activity of antibiotics.
- Multiple metabolic networks within Mtb persisters’ central carbon metabolism and their carbon fluxes are simultaneously shifted to mitigate antibiotic effects.
- Metabolic shifts required to form Mtb persisters are also observed in drug-resistant TB clinical isolates, all of which are a source of new antibiotic targets to efficiently control drug-resistant TB infection.

## Introduction

The introduction of antibiotics into clinical medicine has drastically changed the societal impact of infectious diseases from fatal bacterial infections into predictably curable maladies (Hutchings et al., 2019). The clinical impact of antibiotic treatment, however, has been seriously compromised by the staggering surge of drug resistance (DR), largely due to uncontrolled abuse of antibiotics (Nathan, 2014; Fair and Tor, 2014; Nathan and Barry, 2015; Schrader et al., 2020). DR emergence is exacerbated by the unforeseen failures of antibiotic research. A key factor contributing to this shortfall is a lack of adequate assays to investigate antibiotic effects and their modes of action. Early methods of antibiotic discovery relied on simple screening assays designed to detect phenotypic viability and metabolic activities. However, most antibiotics kill target pathogens through a series of complicated events that cannot be identified by any single assay. Thus, limitations of these classical methods and underdetermined modes of action frequently result in the development of new antibiotics that are highly vulnerable to the emergence of DR mutants because they often fail to eradicate the target pathogens.

The advent of unbiased systems-level disciplines has helped predict DR emergence in clinically isolated pathogens (Chan and Loscalzo, 2012). Metabolomics is the youngest among these techniques and focuses on metabolites present in a certain biological system, such as bacteria after exposure to a given set of environmental conditions (Rhee, 2013). Metabolites are defined as the substrates or products of enzymes and their cofactors that cover diverse cellular activities (Bennett et al., 2009; Lu et al., 2017). Growing evidence has shown that metabolites play a crucial role in the cellular and physiological processes required for the viability and virulence of infectious agents. Metabolomics has become an integral tool to determine the biochemical state of pathogens, thereby elucidating their virulence factors and pathogenicity. The ability to monitor intrabacterial biochemical states and functional interactions with the host immune system has poised metabolomics to uniquely fill the gaps in antibiotic research by investigating metabolic networks required for DR emergence and discovering conceptually new antibiotics (Warner, 2014).

Although tuberculosis (TB) has afflicted humans for more than 4,000 years, it remains a major public health issue in the top 10 leading causes of human death worldwide (Zaman, 2010). Despite recent reduction in the total TB incidence rate, the situation remains extremely dire due to the high frequency of genetic DR mutants (Connolly et al., 2007; Schrader et al., 2020). The World Health Organization (WHO) estimated that, in 2021, roughly half a million new TB cases were caused by DR TB (WHO, 2021). The DR forms of TB include multidrug-resistant (MDR), pre-extensively drug-resistant (pre-XDR), and extensively drug-resistant (XDR). MDR-TB is defined to be resistant to at least isoniazid (INH) and rifampin (RIF), two of the main first-line TB antibiotics. Pre-XDR is newly included to DR TB forms and defined as MDR plus additional resistance to any fluoroquinolone. XDR-TB is defined as MDR plus additional resistance to any fluoroquinolone and at least one additional group A TB antibiotics such as levofloxacin, moxifloxacin, bedaquiline, and linezolid (Viney et al., 2021). These DR TB pose a serious challenge for effective TB control and hinder successful treatment outcomes.

Unlike mutation-acquired DR, *Mycobacterium tuberculosis* (Mtb) persister cells (persisters) possess intrinsic drug tolerance that is phenotypically reversible and free of genetic mutations (Lewis, 2010; Conlon et al., 2016; Fisher et al., 2017). Several groups have recently identified a growing drug-tolerant subpopulation (Adams et al., 2011; Peyrusson et al., 2020). However, we term Mtb persisters as a slowly replicating or non-replicating subpopulation that transiently resists antibiotic effects. Mtb persisters are known to withstand the mycobactericidal effects of nearly all TB antibiotics, even at lethal concentrations, without a change in antibiotic susceptibility upon regrowth under antibiotic-free conditions (Schrader et al., 2021). Accumulating evidence supports that regrowth populations, stemming from bacterial persisters that survive in the human body for a prolonged period of time, are responsible for causing a wide range of chronic and recurrent infections and may serve as a source for permanent DR mutants, especially among immune-compromised patients (Levin and Rozen, 2006; Fridman et al., 2014; Levin-Reisman et al., 2017). Thus, Mtb persisters are a critical, yet seriously understudied, component of the TB disease burden.

Here, we review emerging applications of metabolomics to identify Mtb metabolic and biochemical remodeling during persister formation to inform early TB antibiotic discovery and prevent the development of DR mutations. We focus on central carbon metabolism (CCM) remodeling during Mtb persister formation as the major mechanism of their adaptive strategy and altered biochemical states (Eoh, 2014; Hartman et al., 2017; Jansen and Rhee, 2017). Intrinsic drug tolerance aside, acquired DR is often mediated by heritable genetic mutations. These mutations alter antibiotic activity or their access to cognate antibiotic targets, but recent evidence has suggested that these mutations also arise from specific secondary metabolic remodeling, essential to the physiology for DR mutants (Amato et al., 2013; Derewacz et al., 2013; Hartman et al., 2017). Historically, nearly all clinically relevant TB antibiotics emerged from empirical approaches that have proven to be less effective in killing Mtb persisters. The need for new TB antibiotics has prompted concerted efforts to establish more rational antibiotic discovery platforms. We discuss how metabolomics technology in antibiotic target identification will improve precision and efficacy of the antibiotic development pipeline.

## Main

### The metabolome is a phenotypic fingerprint of biological systems

The genome, transcriptome, and proteome are constructed by polymerization of certain sets of building blocks. Unlike these macromolecules, the metabolome—a collection of metabolites—is not constrained by simple polymerization, which gives rise to vast structural and functional variation and diverse physiological properties (Schwab, 2003; Dettmer et al., 2007; Dunn et al., 2011). Due to this biochemical diversity, there is no single analytic tool to cover the extraction and characterization of all metabolites. Thus, the complete scope of metabolites and their functional interactions remains undefined, which is further complicated by lack of adequate interpretation of analysis. Despite intensive studies, only ~680 metabolites among ~40,000 metabolites present in the Human Metabolome Database (HMDB) have been functionally identified as members of defined pathways (Lu et al., 2017). In studies of Mtb metabolomics, ~300 water-soluble metabolites and ~62 water-insoluble lipids have been identified as the Mtb metabolome (Layre et al., 2011; Eoh and Rhee, 2013; Nandakumar et al., 2014). The extraction rates covering Mtb-specific pathways and the biological relevance of unmapped metabolites remain unknown.

The metabolic phenotype provides a clear readout of Mtb’s biochemical state, as metabolites are the final products of enzymatic, posttranslational, allosteric, and environmental integration under a particular condition (Eoh, 2014; Ehrt et al., 2018). Analysis of intra- and extra-bacterial metabolites in Mtb provides phenotypic information that cannot be accurately deduced by other conventional tools such as genotyping, mRNA profiling, or proteomics. The metabolomics profile presents a unique advantage to alternate technologies by providing a top-down, systems-level outcome that integrates cellular biochemistry, physiological state, and adaptive metabolism required to survive continuously changing environments (Jansen and Rhee, 2017).

The metabolic phenotypes of Mtb persisters are adaptive strategies used not only to regulate replication rates *via* permissive carbon/nitrogen source determination, but also to interact with the host immune system as well as with neighboring populations (Parrish et al., 1998; Dhar et al., 2016). Thus, complete understanding of both metabolite abundances/fluxes and their functional interactions within multiple metabolic networks sheds light on how metabolic state is associated with the level of virulence and tolerance to environmental stresses.

The field of metabolomics has evolved to elucidate these complex metabolic profiles and their corresponding phenotypes (Griffin, 2004). Metabolomics is the youngest systems-level discipline to collectively understand metabolic activities and investigate how metabolic shifts lead to phenotypic optimality, how distinct metabolic activities in Mtb produce different pathogenicity, and how metabolic shifts coordinate adaptive strategies allowing Mtb to survive adverse environments (Jansen and Rhee, 2017). Technically, metabolomics results can be integrated with other systems-level data to improve the understanding of the phenotypic state of an organism (Argelaguet et al., 2018; Subramanian et al., 2020). Recently, Kim et al. integrated metabolomics and transcriptomics profiles of sera from nontuberculous mycobacterial (NTM) disease patients and identified the NTM infection-mediated impact on the host gut microbiome, serum metabolome, and immune defense against NTM infection (Kim et al., 2022). Overlaying the data from other omics studies onto the metabolic landscape is extremely useful to build a quantitative and integrative model of a target biological system. The resulting models have a tremendous potential to produce valuable information connecting metabolic phenotypes to the development of conceptually novel therapeutics and antimicrobial agents.

### Emerging technology of Mtb metabolomics

The metabolomics platform constitutes a multistep process involving culture preparation, metabolic quenching, extraction, sample concentration, detection, data analysis, and interpretation (Wu et al., 2005; Villas-Boas et al., 2005). As metabolic fluxes change within seconds, special harvesting methods are required to avoid artifacts from handling steps before adequate metabolic quenching. Recently, a filter culture-based method was developed to enable non-disruptive handling, rapid quenching, and collection of Mtb cells (Eoh and Rhee, 2013; Nandakumar et al., 2015; Samuels et al., 2017). This method eliminates additional washing and centrifugation steps that could potentially be associated with metabolic changes or losses. Eoh and Rhee and de Cavalho et al. showed that this filter culture-based method supports almost identical Mtb cell wall architecture, growth kinetics, and phenotypic changes even under hypoxic stress conditions as compared to that of conventional liquid culture systems (de Carvalho et al., 2010; Eoh and Rhee, 2013). Accumulating evidence shows that this method also enables reproducible recovery of core metabolites as well as secondary trace metabolites. The unique advantage that this filter culture-based method has over classical liquid cultures is the ease of handling Mtb cells. For example, Eoh et al. modified this method using a procedure termed ^13^C pulse chase labeling to study the shifted carbon fluxes between the CCM and carbohydrate core of cell wall glycolipids (Eoh et al., 2017). Mtb-laden filters were incubated on media containing ^13^C-labeled (U-^13^C) glucose to fully label all CCM intermediates with ^13^C. Then, the filters were transferred to media containing unlabeled glucose to reversely wash only CCM intermediates with unlabeled carbon fraction, ultimately producing Mtb bacilli with unlabeled CCM and a fully ^13^C-labeled carbohydrate core of cell wall glycolipids. This ^13^C pulse chase labeling allowed tracing of glycolipid carbon fluxes during adaptation to various environmental stresses such as hypoxia. The filter culture-based method also allows for simultaneous collection of intracellular metabolites and extracellular secretome. To this end, Eoh and Rhee replaced the underlying agar media with a plastic inset containing chemically equivalent liquid media in direct contact with the underside of the Mtb-laden filters. Growth atop this liquid medium within the inset was indistinguishable from that achieved on adjacent agar medium and enabled timed start–stop measurements of the secretome or antibiotic uptake by sampling cell-free spent liquid media (Eoh and Rhee, 2013). Using this approach, Eoh and Rhee sought to monitor the amount of secreted succinate from hypoxic Mtb. Using a similar method, Luna et al. and Lim et al. separately sought to measure levels of antibiotic uptake by *Acinetobacter baumannii* and Mtb, respectively, after exposure to various environmental stresses such as nutrient starvation or hypoxia (Luna et al., 2020; Lim et al., 2021).

### Mtb persisters are a putative preform of drug-resistant mutants

**Figure 1:**
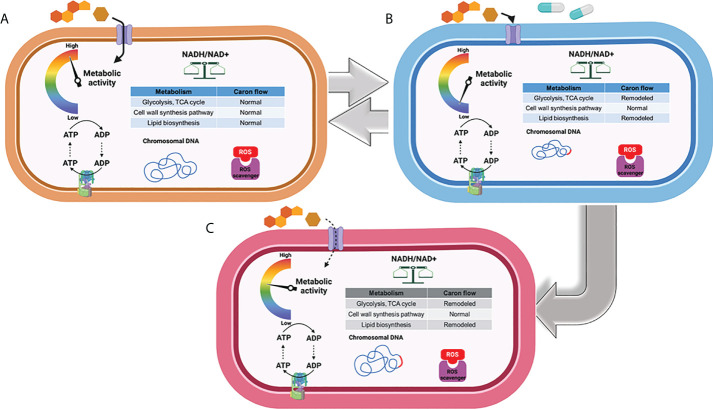
Metabolic phenotype associated with replication, transient drug tolerance, and permanent drug resistance in Mtb. **(A)** Under a replicating state, Mtb maintains the carbon fluxes within its CCM (e.g., glycolysis and the TCA cycle) and cell wall biosynthesis to meet the anaplerotic demands for replication. **(B)** Mtb remodels its metabolic networks in response to environmental stress and antibiotic treatment to produce transient drug tolerance or persistence, notably by rerouting its carbon flux from intermediary metabolism towards the biosynthesis of storage compounds and regulatory molecules. **(C)** Accumulation of ROS as a consequence of antibiotic treatment results in DNA mutagenesis and the development of drug-resistant mutations, leading to the emergence of permanent drug resistance. Figures were created by BioRender.com.

Metabolomics profiles of Mtb offer a unique perspective to define the mechanistic basis underlying persistence and the development of permanent DR mutations. Persistence in latently infected TB patients contributes to survival of Mtb bacilli and may lead to an active form, followed by transmission to healthy individuals (Bryk et al., 2008; Barry et al., 2009; Ehrt and Schnappinger, 2009; Nathan and Barry, 2015; Gold and Nathan, 2017). The phenotypic transitioning between a persister form and an actively replicating form requires growth rate regulation, yet the specific metabolic activities underlying this cell cycle shift remain largely unknown (**Figures 1A, B**).

Whether DR evolves from transiently drug-tolerant Mtb persisters and how Mtb drug tolerance is functionally associated with developing DR mutants are the main subjects of debate in the antibiotic discovery community (Gomez and McKinney, 2004; Fridman et al., 2014; Levin-Reisman et al., 2017; Lewis and Shan, 2017). Accumulating evidence using *Escherichia coli* subjected to intermittent exposure to ampicillin showed that cyclic treatment with the antibiotic delayed its growth lag time—a state of drug tolerance—several cycles ahead of achieving increased minimal inhibitory concentration (MIC), a state of permanent DR against the antibiotic. Delayed lag time after treatment with antibiotics was also detected in Mtb (Levin and Rozen, 2006; Windels et al., 2019b; Saito et al., 2021). Recently, Windels et al. calculated the DR mutation frequency using *E. coli* strains harboring a high or low level of persistence to predict the contribution of bacterial persisters to development of DR by mathematical modeling (Windels et al., 2019a; Windels et al., 2019b). The number of DR colonies emerging from viable cells showed the same correlation, even when a correction for the number of surviving cells was applied. This implies that bacterial persisters formed from high-persistence strains have an increased likelihood to develop DR-conferring mutations, while *E. coli* strains from low-persistence are less likely to mutate to gain DR. The positive correlation between persistence levels and DR mutation rates is an important factor to be considered in new antibiotic discovery, as even minor metabolic remodeling to increase persister formation can be evolutionarily associated with the emergence of DR mutants (Castro et al., 2021). Intriguingly, recent studies have shown that Mtb persisters are also clinically relevant to chronic infections in addition to antibiotic treatment (Zhang et al., 2012; Manina et al., 2015; Sarathy et al., 2018; Schrader et al., 2021). High-persistence forms of Mtb are repeatedly identified in chronic infections and have complicated chemotherapeutic options because they are likely to evolve into MDR-, pre-XDR-, and XDR-Mtb strains (Barry et al., 2009; Lewis, 2010; Nathan, 2014), albeit a controversial view with a significant amount of evidence that refutes this notion (**Figures 1B, C**).

As evidenced from other organisms, Mtb persisters that survive antibiotic effects may have an increased likelihood of acquiring genetic mutations that confer DR due to the subsequent high levels of reactive oxygen species (ROS), an active DNA mutagen (**Figure 1B**) (Davis et al., 2002; Kohanski et al., 2010a; Kohanski et al., 2010b; Long et al., 2016). Thus, the emergence of DR colonies on mycobactericidal antibiotic-containing media over the course of treatment is usually explained by *de novo* DNA mutagenesis, even for Mtb bacilli in a non-replicating state. Spontaneous or antibiotic-induced DNA damage in non-replicating bacilli leads to genetic mutations because, even in the absence of chromosomal replication, these bacilli still display DNA turnover (Cirz et al., 2005; Foster, 2007; Revitt-Mills and Robinson, 2020). Nonetheless, others have proposed that DR mutations in non-growing bacilli are not associated with DNA turnover, but rather stem from a small population of preexisting mutants that grow slowly and give rise to fast-growing mutants that eventually form predominant colonies (Galhardo et al., 2007). *De novo* mutation in non-replicating bacilli can be explained by the gradual accumulation of preexisting DR mutant colonies, shown by the deletion or mutation of several genes that dramatically decreases *E. coli* colony formation on ciprofloxacin-containing media without affecting the number of early colonies or their fitness under growth-restricted conditions (Cirz et al., 2005). Most bacterial pathogens, including Mtb, follow a similar model in which only persisters survive bactericidal antibiotic treatment, forming a viable cell reservoir with an increased likelihood for DR-conferring DNA mutagenesis (Torrey et al., 2016; Windels et al., 2019b; Colangeli et al., 2020).

**Figure 2:**
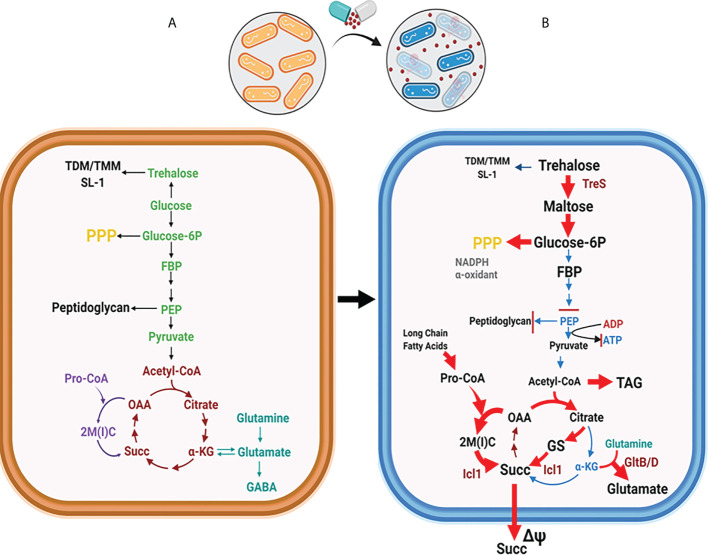
Global metabolic shift in Mtb CCM is required for forming persisters. **(A)** Mtb CCM activities are required for its replication. Enzymatic steps dedicated to glycolysis are depicted in green, the TCA cycle in brown, the MCC in purple, and the nitrogen metabolism in teal. **(B)** Mtb shifts CCM carbon fluxes to form persisters. Rerouted pathways are indicated by red arrows. The trehalose-mediated catalytic shift leads to catabolic remodeling of TDM/TMM, which increases the carbon flux towards the biosynthesis of upper glycolytic intermediates and the PPP, while downregulating the flux to the lower glycolysis. Carbon flow through the glyoxylate shunt and MCC is upregulated to increase the biosynthesis of succinate. Toxic accumulation of MCC intermediates is accompanied by increasing glutamate synthase activity (GltB/D), an activity known to neutralize MCC-mediated metabolic intoxication. Abbreviations are as follows: TMM, trehalose monomycoate; TDM, trehalose dimycolate; FBP, fructose 1,6 bisphosphate; PEP, phosphoenolpyruvate, 2M(I)C, 2-methyl (iso)citrate; OAA, oxaloacetate; αKG, alpha-ketoglutarate; Succ, Succinate; Pro-CoA, propionyl-CoA; GABA, γ-aminobutyric acid; GS, glyoxylate shunt; PPP, pentose phosphate pathway. Figures were created by BioRender.com.

As previously mentioned, antibiotics kill Mtb by accumulation of ROS, which causes irreversible damage on cellular processes essential for Mtb growth and survival. As a countermeasure, Mtb acquires drug tolerance by entering a persistent state which is triggered by metabolic remodeling to mitigate the ROS-mediated cellular damage and subsequently evade mycobactericidal activity (**Figure 2**) (Eoh, 2014; Ehrt et al., 2018). Hence, metabolic remodeling for Mtb persister formation not only sustains its viability but also leads to the accumulation of DR mutations.

### Metabolic remodeling strategies are required for Mtb persister formation

The hallmark of latent TB infection (in a state of persistence) is its ability to survive various antimicrobial environments inside hosts. Mtb replicates even under a fully functioning immune system: soon after recruitment to the site of infection, immune cells release ROS, nitric oxide (NO), and reactive nitrogen intermediates (RNI), and in response, Mtb slows or ceases its growth rates, which enhances drug tolerance levels within the population (Nathan and Hibbs, 1991; Chan et al., 1992; Voskuil et al., 2011). During this process, an adequate immune response will recruit various immune cells that convene into a multicellular structure called a granuloma (Davis and Ramakrishnan, 2009; Ramakrishnan, 2012). The interior of the granuloma is full of biochemical stresses including low oxygen tension (hypoxia), limited access to nutrients (starvation), low pH (acidosis), and accumulation of ROS, RNI, and other toxic metabolites. The majority of viable Mtb bacilli reside within the granuloma in a slowly replicating or non-replicating state by entering a persister form through their metabolic and biochemical shift (Flynn and Chan, 2001; Voskuil et al., 2003; Cosma et al., 2003).

The high level of drug tolerance from non-heritable DR in Mtb persisters is the main factor that destabilizes effective chemotherapies (Lewis, 2010; Zhang et al., 2012; Prax and Bertram, 2014; Sarathy et al., 2018; Gollan et al., 2019). This is largely because most conventional TB antibiotics target cellular processes active only during replication, and thus are no longer effective in killing Mtb persisters. The metabolic shift during persister formation has emerged as a central feature of TB pathogenicity and is fertile ground to identify new antibiotic targets (Levin-Reisman et al., 2017; Windels et al., 2019b).

Mtb CCM plays an essential role in enzymatic transformation of carbon substrates through the activities of glycolysis, gluconeogenesis, the pentose phosphate pathway (PPP), trehalose metabolism, the tricarboxylic acid (TCA) cycle, and TCA cycle-branched pathways such as the glyoxylate shunt and the methylcitrate cycle (MCC) (**Figure 2**) (Baughn and Rhee, 2014; Ehrt et al., 2018). For any organism in an ecosystem, the objective role of their CCM is to meet the stoichiometric demands of sustaining adequate growth rates with optimal efficacy. However, Mtb is microbiologically and metabolically unique in that it is a chronic intracellular pathogen that interacts with humans as its only known host and reservoir (Cosma et al., 2003; Gengenbacher and Kaufmann, 2012). Thus, Mtb bacilli have evolved for survival within the host phagosome as its primary niche. Mtb encounters diverse biochemical stringencies within this adverse environment, many of which play an extrinsic role that triggers the exit from its normal cell cycle, thereby slowing its growth rate and promoting persister formation. Accompanying this phenotypic shift, Mtb persisters alter CCM activities to sustain their viability and slowly replicating or non-replicating physiology (Eoh, 2014; Ehrt et al., 2018). Relieved of the requirement to double its biomass, Mtb persisters have generally been perceived to have low metabolic activities. Indeed, Mtb initiates persister formation by downregulating key component activities in the electron transport chain (ETC), which decreases oxidative phosphorylation and adenosine triphosphate (ATP) biosynthesis to ~10% to that of their replicating counterparts (Eoh and Rhee, 2013; Lim et al., 2021). However, slowly replicating or non-replicating heterogenous persisters face metabolic challenges to preserve the integrity of the cellular components essential for both survival as persisters and re-entry into the normal cell cycle for transmission to new hosts. Metabolic networks of Mtb in a replicating state have been extensively studied, but knowledge of the specific metabolic activities and altered pathways to exit the normal cell cycle is a major area of unmet scientific and medical need.

The absence of classical catabolic carbon repression (CCR) regulatory circuits in Mtb CCM allows the bacilli to co-catabolize glycolytic and gluconeogenic carbon sources simultaneously during replication, a metabolic capacity that confers a survival advantage in an environment where nutrients are scarce (de Carvalho et al., 2010; Borah et al., 2021). This regulatory concept is also true in Mtb persisters. Accumulating evidence using Mtb persisters shows a simultaneous shift in multiple sets of metabolic networks within their CCM (**Figure 2B**) (Eoh and Rhee, 2013; Eoh et al., 2017; Dutta et al., 2019; Lee et al., 2019; Lim et al., 2021; Quinonez et al., 2022). The conventional CCM pattern active during replication was remodeled to favor new catalytic patterns aiming to prolong survival under high levels of environmental stresses by mitigating the metabolic and biochemical toxicities from these stresses. Low NAD^+^ levels act as an intrinsic factor to trigger a metabolic shift during Mtb persister formation, as NAD^+^ is a crucial cofactor involved in a broad range of cellular processes from redox cellular homeostasis to catabolism and energy production (Watanabe et al., 2011; Xie et al., 2020). The absence of lactate dehydrogenase in Mtb metabolic networks causes ETC-mediated oxidative phosphorylation to be strictly essential for virulence and viability (Serafini et al., 2019). The reliance of Mtb virulence on the ETC was supported by the clinical discovery of a new second-line TB antibiotic, bedaquiline (BDQ), with F1F0 ATP synthase of the Mtb ETC as its target (Mahajan, 2013; Kundu et al., 2016). Antibiotics targeting the Mtb ETC are known to be effective against DR TB strains and have potential to shorten TB treatment duration (Rao et al., 2008). For example, the *qcrB* gene of the cytochrome bc1:aa3 complex is the target of Q203, another novel ETC inhibitor (Pethe et al., 2013; Harrison et al., 2019). Intriguingly, Kalia et al. recently reported that the potency of Q203 is determined by CCM shifts that respond to available carbon sources (Kalia et al., 2019). Catalytic activity for glycerol had a positive impact on Q203 antibiotic potency, as the cytochrome bd terminal oxidase became functionally important while consuming glycerol as a carbon source. The potency of BDQ is also affected by CCM shifts and available carbon sources (Wang et al., 2019; Mackenzie et al., 2020). These findings emphasize the potential of Mtb persister metabolic activities for new antibiotic discovery.

Mtb persisters collected after exposure to biochemical growth-limiting stresses that resemble the host environment or from the sputum of active pulmonary TB patients have both long been popular models used to understand the adaptive strategies of Mtb. However, these findings were focused on transcriptional analysis due to suboptimal culture methods and analytical tools. Only recently has research begun to focus on the metabolism of intrinsic drug tolerance. The following are reported examples of metabolic networks shifted during Mtb persister formation.

#### Metabolic shift from the TCA cycle to TAG biosynthesis

**Figure 3:**
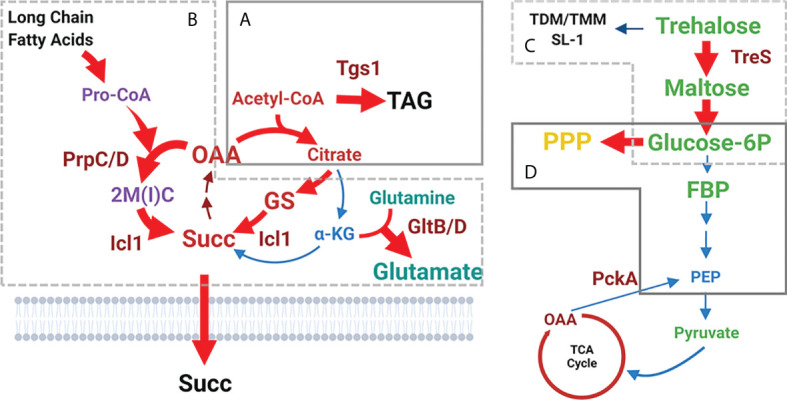
Shifted catalytic activities of Mtb persister CCM. **(A)** Acetyl-CoA-mediated carbon flux shift towards the biosynthesis of triacylglycerol (TAG). Consequently, the flux towards the oxidative arm of the TCA cycle is downregulated. **(B)** Reduced carbon flux towards the oxidative arm of the TCA cycle is further facilitated by increasing the carbon flux through the glyoxylate shunt to biosynthesize succinate. Simultaneously, MCC activity is upregulated, resulting in toxic accumulation of MCC intermediates including propionyl-CoA. GltB/D-mediated conversion from glutamine to glutamate is activated as a strategy to neutralize MCC toxicity. **(C)** Trehalose-mediated carbon shift from the cell wall glycolipids towards the biosynthesis of upper glycolytic intermediates such as glucose 6P. **(D)** Glucose 6P-mediated carbon flux shift from downstream glycolysis towards the biosynthesis of PPP intermediates. Abbreviations are the same as depicted in **Figure 2**. Figures were created by BioRender.com.

Baek et al. identified the *tgs1* gene using transposon (Tn) mutagenesis while screening for genes whose Tn insertion resulted in Mtb growth or survival advantage under hypoxic stress (Baek et al., 2011). Tgs1 is characterized as a triacylglycerol (TAG) synthase in Mtb and is catalytically involved in condensing glycerol 3-phosphate and acyl-CoA. As Tgs1 is responsible for the dominant TAG biosynthetic activity in Mtb under hypoxia, it is required for optimal virulence within host niches. Targeted deletion of *tgs1* in Mtb (Δtgs1) allows bacilli to continue replication even under hypoxia, indicating the essential role of Tgs1 for the slowly replicating or non-replicating phenotype of Mtb persisters. Metabolomics analysis of Δtgs1 showed that acetyl-CoA-mediated carbon flux towards the biosynthesis of TAG was prevented, with its flux redirected towards the biosynthesis of intermediates in the oxidative arm of the TCA cycle including citrate and α-ketoglutarate (α-KG). This pathway is a major biosynthetic source of NADH, an initial substrate of Mtb ETC. As a result, the carbon flux shift in Δtgs1 significantly increases antibiotic susceptibility against a broad range of clinically relevant antibiotics [e.g., INH, streptomycin (STR), ciprofloxacin (CPF), and ethambutol (EMB)] even under persister-inducing environments and within mouse models. The mechanistic basis underlying the antibiotic susceptibility of Δtgs1 was supported by comparing the drug tolerance and growth pattern of Mtb overexpressing citrate synthase (CitA), an enzyme that condenses oxaloacetate (OAA) and acetyl-CoA in the oxidative arm of the TCA cycle. This study indicates that environmental stress-induced rewiring of acetyl-CoA-mediated carbon flux is an adaptive response of Mtb to environmental stress used to regulate its growth rate and drug tolerance (**Figure 3A**).

Consistent with the foregoing findings from Δtgs1, accumulation of intrabacterial TAG may act as a metabolic biosignature associated with reduced growth rates and increased drug tolerance *in vitro* and in mouse models (Daniel et al., 2011). Mtb expresses the TAG efflux pump, LprG (Rv1410), to adjust intrabacterial TAG levels by pumping TAG out of the cytoplasm (Farrow and Rubin, 2008; Martinot et al., 2016). TAG accumulation within lipid droplets in the cytoplasm of *lprG*-deficient Mtb (ΔlprG) led to reduced growth rates accompanied by drug tolerance. TAG, located at the outer leaflet of the outer membrane, functions as a structural barrier while also providing a source of ATP *via* the β-oxidation pathway of TAG acyl-chains. Thus, intrabacterial TAG levels are metabolically associated with bacterial ATP biosynthesis, another biosignature for bacterial persisters. Like acyl-chains of phthiocerol dimycocerosates (PDIM) (Lee et al., 2013), TAG serves as a catalytic sink to alleviate the accumulation of toxic propionyl-CoA, an initial MCC substrate. Within hypoxic and lipid-rich macrophages, Mtb often uses host fatty acids derived from macrophage TAG acyl-chains after β-oxidation to build up its own TAG stores. Thus, the catalytic shift required for synthesis, accumulation, translocation, and distribution of TAG appears to be critical for cellular homeostasis in Mtb persisters’ optimal metabolic state.

#### Metabolic shift from the oxidative arm of the TCA cycle to the glyoxylate shunt

Recent observations have identified isocitrate lyase (ICL) as a specific mediator to produce the metabolic shifts of Mtb persisters (Eoh and Rhee, 2013; Nandakumar et al., 2014). ICL has been identified as an essential enzyme for Mtb persister formation and high drug tolerance *via* its new role in antioxidant defense rather than its canonical role in fatty acid metabolism. Work by Nandakumar et al. showed that Mtb changes its carbon fluxes through the ICL-mediated glyoxylate shunt as a method to evade antibiotic effects (Nandakumar et al., 2014). In this study, Mtb enhanced its survival rates in response to mycobactericidal concentrations of clinically relevant first-line TB antibiotics such as INH, RIF, and STR, by increasing carbon flux through the glyoxylate shunt and bypassing the oxidative arm of the TCA cycle. Accumulating evidence has shown that this metabolic shift produces glycine and succinate as metabolic end products without overproducing NADH and ROS (Eoh and Rhee, 2013). *icl*-deficient Mtb (Δicl) became hypersensitive to treatment with INH, RIF, and STR, and the normal level of antibiotic sensitivity was restored by supplementation with a chemical antioxidant such as thiourea. These findings collectively suggest that the catalytic shift from the canonical TCA cycle towards biosynthesis of glyoxylate shunt intermediates serves as an adaptive strategy used to evade antibiotic effects. This is accomplished by mitigating the production of respiratory radicals arising from canonical TCA cycle-mediated NADH production and ETC activity (**Figure 3B**).

Under a physiological context, Giffin et al. explored possible roles of glycine biosynthesis by characterizing a glycine dehydrogenase (Ald)-deficient Mtb (Δald) (Giffin et al., 2016). First, they found that in addition to *icl*, both the mRNA expression of *ald* and the enzymatic activity of its translated product were increased while entering the hypoxia-induced persister state. Intriguingly, Δald showed minor survival disadvantages under *in vitro* hypoxia, but a significant lag phase delay upon resuming growth after reoxygenation. Ald is proposed to maintain optimal NAD^+^ recycling during initial regrowth after reoxygenation. Two independent studies separately identified an essential role of succinate metabolism in the ability of Mtb persisters to survive hypoxic stress (Watanabe et al., 2011; Eoh and Rhee, 2013). Watanabe et al. found a considerable increase in extracellular succinate from Mtb persisters, which is catalytically linked to the reverse TCA cycle activity, identified by stable ^13^C isotope tracing metabolomics using [U-^13^C] glucose. This rearrangement of succinate metabolism allows Mtb persisters to maintain an energized membrane potential, despite the lack of a primary ETC terminal electron acceptor, by the reductive branch activity of the TCA cycle followed by accumulation and secretion of succinate. Unexpectedly, knockout of the canonical fumarate reductase in Mtb (Δfrd) was not functionally associated with reduced succinate secretion and impaired membrane potential. Eoh and Rhee used stable ^13^C isotope tracing metabolomics of Mtb persisters under hypoxia using [U-^13^C] acetate. This work showed that, upon exposure to hypoxia, Mtb persisters slowed TCA cycle activity and increased succinate production *via* ICL in the glyoxylate shunt. By fermenting fumarate to succinate, Mtb persisters are capable of sustaining both membrane potential and ATP synthesis under low oxygen levels (**Figure 3B**).

In Mtb persisters, succinate biosynthesized by either the reverse reductive branch of the TCA cycle or the glyoxylate shunt is further catalyzed by succinate dehydrogenase (Sdh1), the enzyme that couples growth processes controlled by the TCA cycle with energy production resulting from the ETC. Hartman et al. characterized the impact of genetically deleting the membrane anchor subunit of Mtb Sdh1 (Δsdh1). Sdh1 oxidizes succinate to biosynthesize fumarate and deliver two electrons to ETC membrane electron carriers such as menaquinone (Hartman et al., 2014). Stable ^13^C isotope tracing metabolomics of Δsdh1 confirmed that succinate oxidation was downregulated during aerobic growth but uncontrolled during adaptation to hypoxic conditions. As a result, Δsdh1 failed to survive during the stationary phase or during chronic infection in the C3HeB/FeJ mouse model due to several impaired bioenergetic activities arising from a mismatch between glyoxylate shunt-mediated succinate biosynthesis and ETC respiration.

These studies identified enzymes involved in the glyoxylate shunt and succinate metabolism—malate dehydrogenase, succinate dehydrogenase, and ICL—as potential sources of new drug targets to eradicate Mtb persisters.

#### Metabolic shift from cell wall glycolipids to glycolysis

Eoh et al. showed that the adaptive metabolic shift of Mtb persisters under hypoxic conditions was mediated by an accumulation of upper portion glycolysis intermediates, such as glucose 6-phosphate (Glc 6P) and fructose 1, 6-bisphosphate (FBP) (Marrero et al., 2013; Eoh et al., 2017). This resulted in a subsequent depletion in phosphoenolpyruvate (PEP), the most downstream glycolysis intermediate, and upstream trehalose, a glucose disaccharide (Eoh et al., 2017; Lim et al., 2021). These changes were found to be caused by catabolic remodeling of cell wall glycolipids such as trehalose monomycolate (TMM) and trehalose dimycolate (TDM) *via* trehalose synthase (TreS) activity. A *treS*-deficient Mtb (ΔtreS) was unable to adapt to, or recover from, incubation under hypoxia stress. These results were similarly reproduced by treatment with validamycin A, a validated chemical inhibitor of TreS. These changes were dissociated from TCA cycle remodeling, as confirmed by inclusion of NO_3_, a physiologically relevant terminal electron acceptor of ETC capable of supporting near-aerobic levels of canonical TCA cycle activity under hypoxic conditions (Eoh and Rhee, 2013) (**Figure 3C**).

As alluded, Lee et al. supported the crucial role of the trehalose-mediated catalytic shift as an adaptive strategy of Mtb persisters used to survive many clinically relevant biochemical stresses and antibiotic effects (Lee et al., 2019). This study used an *in vitro* biofilm culture to mimic host-implemented stresses (e.g., hypoxia, nutrient starvation, and toxic metabolites) and to trigger persister formation. Mtb persisters were generated in the biofilm culture in pelleted form at both the wall–media border and air–media interface (Ojha et al., 2005; Kulka et al., 2012; Ojha et al., 2015). Floating and precipitated fractions, which contain replicating and dead bacilli, were removed to selectively collect Mtb culture enriched with persister bacilli. As shown in Mtb persisters under a hypoxic condition, metabolomics analysis of Mtb persisters collected from the biofilm culture showed decreased levels of TMM, TDM, UDP-glucose, and maltose phosphate, which reciprocally matched increases in maltose, Glc 6P, and FBP (Lee et al., 2019). This metabolomics profile differs from that of replicating Mtb, which manages trehalose metabolism for the biosynthesis of the cell wall glycolipids such as TMM and TDM. As glycolysis is a metabolic source of ATP biosynthesis *via* substrate level phosphorylation, the trehalose-mediated catalytic shift serves as an alternate source of ATP for Mtb persisters with a functionally downregulated ETC. Indeed, as seen in ΔtreS or treatment with validamycin A, inhibition of the trehalose-mediated catalytic shift renders Mtb hypersensitive to nearly all TB antibiotics including INH, RIF, and BDQ. Metabolomics profiling of ΔtreS revealed that this hypersensitivity was likely due to ROS damage, and co-treatment with thiol-based antioxidant thiourea afforded a roughly 1.5 log_10_ increase in colony-forming units (CFUs), approaching nearly complete protection against second-line TB antibiotics such as BDQ. These findings indicate that the trehalose-mediated catalytic shift is an adaptive strategy to primarily sustain ATP levels and additionally serve as a source of antioxidant biosynthesis to protect Mtb persisters from antibiotic-induced antimicrobial damage.

Recently, Ruhl et al. reported that another set of glycolipids in Mtb, termed sulfolipid-1 (SL-1), plays a role as a nociceptive neuron activating molecule (Ruhl et al., 2020). The host cough reflex was significantly decreased during infection with an Mtb genetic mutant lacking SL-1, suggesting that Mtb persisters take advantage of this reflex to transmit to new healthy individuals. The structure of SL-1 contains a trehalose 2-sulfate core that is esterified with four acyl-chains. As trehalose is present in its carbohydrate core, the SL-1-mediated cough reflex may be reciprocally related with the level of foregoing trehalose-mediated catalytic shift activity (**Figure 3C**). During latent infection, where Mtb mostly resides in its persister form, the trehalose-mediated catalytic shift activity is enhanced to induce trehalose consumption towards the biosynthesis of glycolysis intermediates. This carbon-flux shift may result in SL-1 downregulation within the cell wall; TB patients with a latent TB infection express weaker SL-1-mediated cough reflex. When exiting from a persister state by resuming its growth, Mtb stops the trehalose-mediated catalytic shift by restoring its flux towards the biosynthesis of cell wall glycolipids including SL-1, which triggers the host cough reflex for immediate transmission.

#### Metabolic shift from glycolysis towards the biosynthesis of PPP intermediates

Shi et al. proposed an interesting model in which Mtb persisters reroute their carbon and nitrogen fluxes from intermediary metabolism involved in energy production and macromolecule biosynthesis towards the biosynthesis of storage compounds such as TAG or glutamate (Shi et al., 2010). This adaptive metabolism was observed in response to *in vitro* growth arrest or *in vivo* mouse lung infection. Lim et al. recently reported that hypoxia-induced TAG biosynthesis is mediated by a metabolic shift involving downregulation of glycolytic intermediates such as PEP (Lim et al., 2021). Previous metabolomics findings revealed that accumulation of Glc 6P and FBP was kinetically unmatched to that of PEP, indicating disproportionate glycolysis activity (Eoh et al., 2017; Lee et al., 2019; Lim et al., 2021). PEP depletion in Mtb persisters is functionally associated with limited biosynthesis of oxaloacetate (OAA), a substrate of CitA activity, resulting in accumulation of acetyl-CoA and reciprocal induction of acetyl-CoA-mediated carbon flux towards the biosynthesis of TAG. Therefore, enhanced TAG biosynthesis is catalytically associated with downregulation of the lower portion of glycolysis (**Figure 3D**).

Work by Lee et al. found that reduced carbon flux from Glc 6P to lower glycolysis intermediates is reciprocally related with enhanced carbon flux towards the biosynthesis of PPP intermediates. The PPP is a metabolic pathway parallel to glycolysis conserved in all biological systems and represents the first committed step of glucose metabolism by sharing Glc 6P as a common substrate (Stincone et al., 2015). The PPP intermediates—ribose 5-phosphate (ribose 5P) and sedoheptulose 7-phosphate (sedoheptulose 7P)—function as biosynthetic sources of nucleic acids, histidine, and nicotinamide-adenine dinucleotide phosphate (NADPH) (Lee et al., 2019). The PPP serves a critical role in supporting some cancer cell survival and growth by providing NADPH and generating ribose 5P for nucleic acid biosynthesis (Tozzi et al., 2006; Riganti et al., 2012; Patra and Hay, 2014). NADPH is a cofactor required for the biosynthesis of fatty acids and acts as an electron donor for the biosynthesis of γ-glutamylcysteine, which is involved in ROS scavenging (Blokhina et al., 2003). This study claimed that accumulated Glc 6P can act as the preferred carbon source for biosynthesis of PPP intermediates and not for lower glycolysis intermediates. Intriguingly, an Mtb mutant (ΔdevB) lacking the second enzyme in the PPP, 6-phosphogluconolactonase (DevB), was still able to form persisters at levels similar to those of wild-type Mtb in an *in vitro* biofilm culture (Lee et al., 2019). Together, these findings suggest that the catalytic shift from Glc 6P towards the biosynthesis of PPP intermediates is another metabolic strategy of Mtb persisters used to meet their anabolic demands and combat oxidative stresses (**Figure 3D**).

A gene termed *pckA* encodes the enzyme PEP carboxykinase (PEPCK), which catalyzes the first committed step in Mtb gluconeogenesis (Liu et al., 2003; Marrero et al., 2010). Transcriptional silencing of *pckA* led to Mtb clearance in mice during the chronic phase of infection. In conjunction with the evidence showing the importance of PEPCK’s canonical role in fatty acid metabolism and PEP biosynthesis, mouse infection models implicated an essential role in Mtb gluconeogenesis for their persister physiology. However, the impaired survival of the *pckA* knockout strain (ΔpckA) could not be recapitulated when culturing ΔpckA on fatty acid media or in resting or immune-activated mouse bone marrow-derived macrophages (BMDMs) (Marrero et al., 2010). Similar to Δicl, growing evidence has identified PEPCK as one determinant required for the Mtb metabolic shift, but has failed to clarify its metabolic role because of the inaccuracy in its catalytic annotations and *in vitro* biochemical studies. Separate studies demonstrated that Mtb PEPCK could catalyze the reverse reaction in an anaplerotic, not gluconeogenic, direction when Mtb was exposed to a reducing environment as seen in hypoxic conditions (Machova et al., 2014). Computational modeling of catalytic shifts has similarly suggested that Mtb PEPCK may execute in an anaplerotic direction during intracellular growth within THP-1 macrophages. Unlike these findings, Lim et al. conducted stable ^13^C isotope tracing metabolomics using ΔpckA under a hypoxic condition in the presence of [U-^13^C] acetate and found that there were no significant differences in the abundance and ^13^C enrichment rates for the majority of Mtb CCM intermediates such as the reductive arm of the TCA cycle (e.g., OAA as a substrate of PEPCK and malate) and PEP (a product of PEPCK) as compared to those of wild-type Mtb under the same condition (Lim et al., 2021). This metabolomics profile implies that PEP depletion in Mtb persisters is attributed to downregulation of gluconeogenic carbon flux as a result of defective PEPCK activity in both gluconeogenic and anaplerotic directions.

#### Induced metabolic fluxes through the MCC

Environmental stress induces catalytic shifts that are associated with altered TCA cycle activity by enhancing the carbon flux through the reductive arm of the TCA cycle while concomitantly downregulating flux through the oxidative arm. This discontinued TCA cycle activity is catalytically attributed to enhanced activity through the MCC, a pathway that branches from the reductive arm of the TCA cycle. Eoh and Rhee conducted stable ^13^C isotope tracing metabolomics using Δicl in the presence of [U-^13^C] acetate or [U-^13^C] propionate as a single carbon source and confirmed that ICL also functions as a methylisocitrate lyase (MCL), the last enzyme in the MCC, which catabolizes propionate after β-oxidation of odd-chain fatty acids (Eoh and Rhee, 2014). Δicl was shown to be incapable of establishing infection in a mouse model of pulmonary TB (McKinney et al., 2000; Munoz-Elias and McKinney, 2005). Loss of MCL activity resulted in accumulation of propionyl-CoA and MCC intermediates, providing a mechanistic basis underlying the Δicl bactericidal phenotype in propionate media or a chronic infection mouse model (Gould et al., 2006). Recent metabolomics studies of Mtb persisters showed that the MCC and the reductive arm of the TCA cycle are two top pathways greatly altered during adaptation to antibiotic treatment (Eoh and Rhee, 2013; Jansen and Rhee, 2017). Quinonez et al. supported this finding by showing that Mtb cultured in media containing short-chain fatty acids (e.g., either propionate or acetate) form a significantly greater portion of antibiotic-induced Mtb persister bacilli than those cultured in media containing glycerol or glucose due to the buildup of MCC and reductive TCA cycle intermediates (Quinonez et al., 2022). Collectively, MCC remodeling in the fatty acid-induced persister state of Mtb is yet another adaptive strategy to achieve improved levels of drug tolerance (**Figure 3B**).

Despite the phenotypic advantage functionally associated with high level of drug tolerance, MCC remodeling is often accompanied by accumulation of toxic intermediates such as acetyl-CoA, propionyl-CoA, or MCC intermediates (Savvi et al., 2008; Eoh and Rhee, 2014; Puckett et al., 2017). Biological systems always employ adaptive strategies, including catalytic activities, required for importing new host nutrients and degrading these carbon sources into small compounds that can be further catabolized. As part of their adaptive strategies, altered metabolic networks of Mtb persisters also include the ability to neutralize adverse metabolic effects from new carbon catabolism. To elucidate this activity, Lee et al. compared metabolic networks between virulent Mtb and the attenuated Bacillus Calmette-Guérin (BCG) vaccine strain to propose that BCG lacks the same adaptive strategies like glutamate synthase (GltB/D)-mediated activity to convert glutamine to glutamate as a metabolic effort to neutralize propionate and MCC-induced metabolic intoxication (Lee et al., 2018). Thus, the functional crosstalk between active MCC and GltB/D-mediated metabolic neutralization is essential for a high level of drug tolerance of Mtb persisters. Inactivation of this crosstalk will selectively kill Mtb persisters by maximizing the effect from propionate and MCC-meditated metabolic intoxication, providing a source of new antibiotic targets (**Figure 3B**).

Collectively, conventional TB treatment is not effective in killing Mtb persisters and preventing the development of DR Mtb mutants, as the current regimen targets only the metabolic activities required for replication. Slowly replicating or non-replicating Mtb persisters alter their metabolic networks by remodeling the carbon fluxes previously mentioned. It is critical to map the holistic metabolic fluxes altered while forming Mtb persisters and pinpoint the catalytic enzymes or pathways required to produce these carbon fluxes as a source of new drug targets.

### Metabolic shifts shared by *Mtb* persisters and drug-resistant mutants

Even in developed countries including the USA, treating DR TB patients takes ~24 months and ~$393,000 per case, which is substantially more time-consuming and expensive than ~6 months and ~$49,000 for a drug-sensitive TB patient (Schrader et al., 2020). Thus, the major focus of our TB treatment campaign and research is on preventing the emergence of DR through the elucidation of metabolic activities necessary to sustain the metabolism and viability of DR Mtb strains. One possible mechanism suggests that exposure of drug-sensitive Mtb strains to bactericidal concentrations of TB antibiotics can select for Mtb persisters, during which permanent DR Mtb mutants evolve (Bifani et al., 2008; Brauner et al., 2016; Goossens et al., 2020).

Intriguingly, the metabolic shifts required to form Mtb persisters are also often detected in DR TB clinical isolates. Recently, Lim et al. showed that supplementing Mtb persisters with PEP under a hypoxic condition significantly reduced antibiotic-induced persister formation and suggested that metabolic remodeling from PEP depletion plays an important role in shifting Mtb persister metabolism. Intriguingly, this report also found that PEP supplementation significantly reduced the DR mutation rate against RIF, confirming a functional linkage between metabolic remodeling of Mtb persisters and the emergence of DR Mtb mutants (Lim et al., 2021). Metabolomics profiles of DR TB clinical isolates supported this notion, as PEP abundance and PEP/pyruvate ratio were significantly lower in MDR- and XDR-TB clinical isolates than in drug-sensitive clinical isolates (Lim et al., 2021). This indicates that the metabolic shift arising from accumulation of upper glycolysis intermediates, followed by low levels of PEP, is advantageous not only for Mtb persisters but also for the emergence of genetic mutation-mediated DR Mtb mutants.

Two comprehensive genetic studies screened for Mtb genes that are functionally altered to avoid antibiotic effects using DR TB clinical isolates (Bellerose et al., 2019; Safi et al., 2019). Both identified transient frameshift mutations in the hypervariable homopolymeric region of glycerol-3-kinase (GlpK), an enzyme required for optimal glycerol catabolism and associated with tolerance against INH, RIF, pyrazinamide, and moxifloxacin. Loss of GlpK function was shown to be a specific metabolic marker of DR TB clinical isolates. These studies hypothesized that the *glpK* frameshift mutations resulted in drug tolerance through metabolic changes that reduced growth and promoted expression of stress response regulators, such as DosR and SigH, thus diminishing antibiotic effectiveness. This stress response also induced genes related to TAG biosynthesis. In addition, DR TB clinical isolates exhibit a catabolic defect in glycerol consumption due to a lack of functional GlpK that may alter global glycolysis activity, presumably facilitating the depletion of PEP (Lim et al., 2021). Investigations on the effects of PEP supplementation on antimicrobial synergy with second-line TB antibiotics against DR TB clinical isolates are warranted.

Disproportionate activities within glycolysis of DR TB clinical isolates were also identified in a study reported by Lee et al. by collecting and characterizing three drug-sensitive and six DR TB clinical isolates (Lee et al., 2019). The growth of DR TB clinical isolates was impaired in media containing carbohydrates as a single carbon source, while drug-sensitive TB clinical isolates grew at a rate similar to that of the H_37_Rv lab strain. Butyric acid (a C_4_ short-chain fatty acid) supported the growth of both drug-sensitive and DR TB clinical isolates at a similar level. *In vitro* growth patterns of all TB clinical isolates in media containing butyric acid were enhanced by supplementation with trehalose. Intriguingly, the improved growth of DR TB clinical isolates was suppressed by treatment with validamycin A or a trehalose analog, 6-azido-6-α,α’-trehalose (6-treAz), while the enhanced growth of drug-sensitive TB clinical isolates was relatively unaffected even after treatment with validamycin A. The specific sensitivity of DR TB clinical isolates to treatment with a TreS-specific inhibitor indicated that DR TB clinical isolates, unlike drug-sensitive clinical isolates, rely on TreS activity in managing their CCM activities. These findings were further confirmed by metabolomics and lipidomics profiling. Chemical inhibition of the trehalose-mediated catalytic shift afforded more rapid and vigorous depletion in CFUs of DR TB clinical isolates against BDQ by ameliorating its weak early bactericidal activity (Koul et al., 2014; Lee et al., 2019). These results indicate that the trehalose-mediated catalytic shift can serve as an adjunctive target option in combination with second-line TB antibiotics for treatment of DR TB infections.

Although drug tolerance is a reversible DR state, growing evidence indicates that the presence of specific genetic mutations is also associated with the propensity of Mtb to enter an drug-tolerant persister state (Goossens et al., 2020). Hicks et al. used a genome-wide study of TB clinical isolates collected in China to identify previously unappreciated bacterial intrinsic factors that accelerate the development of DR mutations (Hicks et al., 2018; Hicks et al., 2019). This study showed that many DR TB clinical isolates harbor genetic mutations at the gene encoding the transcriptional factor *prpR*. The mutations at *prpR* conferred conditional drug tolerance against some TB antibiotics such as RIF, INH, and ofloxacin, by altering the main propionate metabolism pathways including the MCC and methylmalonyl-CoA pathway. Indeed, *prpR* is a transcriptional factor that is divergently transcribed from the *prpDC* operon in Mtb, which encodes the first two enzymes in the MCC. Intriguingly, *prpR*-mediated drug tolerance is carbon source-dependent *in vitro*. Consistent with previous findings, this conditional drug tolerance depends on metabolic intoxication arising from accumulation of propionyl-CoA and MCC intermediates (Eoh and Rhee, 2014; Lee et al., 2018). Thus, as discussed by Lee et al., the GltB/D-mediated metabolic shifts that presumably function to neutralize foregoing MCC intermediates and propionyl-CoA mediated toxicity should be studied as a new antibiotic target to kill Mtb persisters and DR TB infection by maximizing the level of metabolic intoxication.

High persisting (*hip*) mutants of genes in fatty acid metabolism and CCM have been isolated after treatment with bactericidal concentrations of RIF and STR (Torrey et al., 2016). The *hip* mutants with reduced fatty acid degradation activities, such as those catalyzed by FadE30, are likely to manifest a reduction in fatty acid metabolism. This is consistent with the metabolic shift of carbon fluxes away from the TCA cycle in favor of the biosynthesis of long-chain fatty acids. The upregulation of *icl* and *tgs1* in a condition lacking wild-type *hip* supports the foregoing metabolic examples seen in drug-tolerant Mtb persisters.

## Conclusion

Metabolic remodeling strategies serve as an intrinsic virulence factor of Mtb through multiple roles beyond nutrient uptake and consumption required for increasing its biomass. These roles include cellular intrinsic processes such as carbon flux homeostasis, membrane bioenergetics, antioxidant defense, and cell cycle remodeling, as well as extrinsic factors such as regulation of immunoreactivity, drug tolerance, and DR. Thus, new antibiotic discovery should be implemented by elucidating such roles, as metabolic shifts are the biochemical foundation of all physiological processes (Miethke et al., 2021). It is of particular importance to elucidate phenotype-specific metabolic shifts and vulnerability to condition-specific modulation that explain the diverse roles of metabolic remodeling in the pathogenicity of Mtb persisters. The advent of new technologies has made it possible to revise old-fashioned identification of new antibiotic targets based on structure–activity relationship (SAR) or high-throughput screening. Metabolomics-based antibiotic discovery combined with complete knowledge about the Mtb persister metabolic shifts promises to not only expand our specific knowledge about TB pathogenicity, but also enable intelligence technology-based computational simulations to address more complex questions that go beyond reconstruction of drug to target interactions. In the new antibiotic discovery platform, knowledge about metabolic remodeling specific to host-induced persistence and drug tolerance remains considerably important to identify a more effective TB drug regimen and shorten its duration.

## Author contributions

HE, RL, and JL participated in conceptualization and writing of the original draft. JJL and PS substantially contributed to the discussion of the content, review, and editing. All authors contributed to the article and approved the submitted version.

## Funding

This work was supported by NIH (R21AI139386 and R56AI143870), American Lung Association Innovation Award, and Donald E. & Delia B. Baxter Foundation to HE.

## Acknowledgments

We would like to thank all Eoh lab members for reading and commenting on the manuscript. We are grateful to C. Stallings for kindly sharing her unpublished manuscript. We apologize that many papers could not be discussed owing to the lack of space.

## Conflict of interest

The authors declare that the research was conducted in the absence of any commercial or financial relationships that could be construed as a potential conflict of interest.

## Publisher’s note

All claims expressed in this article are solely those of the authors and do not necessarily represent those of their affiliated organizations, or those of the publisher, the editors and the reviewers. Any product that may be evaluated in this article, or claim that may be made by its manufacturer, is not guaranteed or endorsed by the publisher.
